# Nanoscale observation of surface potential and carrier transport in Cu_2_ZnSn(S,Se)_4_ thin films grown by sputtering-based two-step process

**DOI:** 10.1186/1556-276X-9-10

**Published:** 2014-01-08

**Authors:** Gee Yeong Kim, Ju Ri Kim, William Jo, Dae-Ho Son, Dae-Hwan Kim, Jin-Kyu Kang

**Affiliations:** 1 Department of Physics, Ewha Womans University, Seoul 120-750, Korea; 2 Advanced Convergence Research Center, Daegu Gyeongbuk Institute of Science and Technology (DGIST), Daegu 711-873, Korea

**Keywords:** Cu_2_ZnSn(S,Se)_4_, Cu(In,Ga)Se_2_, Kesterite, Conductive atomic force microscopy, Kelvin probe force microscopy

## Abstract

Stacked precursors of Cu-Zn-Sn-S were grown by radio frequency sputtering and annealed in a furnace with Se metals to form thin-film solar cell materials of Cu_2_ZnSn(S,Se)_4_ (CZTSSe). The samples have different absorber layer thickness of 1 to 2 μm and show conversion efficiencies up to 8.06%. Conductive atomic force microscopy and Kelvin probe force microscopy were used to explore the local electrical properties of the surface of CZTSSe thin films. The high-efficiency CZTSSe thin film exhibits significantly positive bending of surface potential around the grain boundaries. Dominant current paths along the grain boundaries are also observed. The surface electrical parameters of potential and current lead to potential solar cell applications using CZTSSe thin films, which may be an alternative choice of Cu(In,Ga)Se_2_.

**PACS number:** 08.37.-d; 61.72.Mm; 71.35.-y

## Background

Cu_2_ZnSn(S,Se)_4_ (CZTSSe) quaternary semiconductors attract a lot of interest for thin-film solar cells [1]. Competition in the solar cell market is nowadays hard-hitting, so it is getting more concern on the cost in the manufacturing of the thin-film solar cells. CZTSSe consists of relatively cheap and earth-abundant elements of Zn and Sn. In contrast, Cu(In,Ga)Se_2_ (CIGS), which is now mostly promising for commercialization, has expensive and rare elements of In and Ga. CZTSSe shows high absorption coefficient and the band gap of it can be tuned with changing S and Se composition.

So far, the highest conversion efficiency of CZTSSe is reported as 11.1% in non-vacuum process with hydrazine [2] and 9.2% in vacuum process by co-evaporation [3,4]. Very recently, Solar Frontier announced the conversion efficiency of 10.8% in the CZTSSe solar cell module of 14 cm^2^[5], which indicates presumably 12 to 13% of the conversion efficiency in the cell level. For large area deposition, sputtering methods have an advantage in production of CZTS-based solar cells [6,7]. It is likely that compound sources such as ZnS and SnS can improve adhesion between the substrate and the thin film during deposition. Moreover, it is believed that the method can increase grain size, control composition, and improve surface morphology of precursors [8,9]. In order to put Se into the as-grown CZTS stacked precursors, optimization of annealing conditions of the precursors in Se atmosphere is decisively important. In previous reports, the different stacking orders of precursors determine the crystallinity and grain growth of the CZTSSe thin films [10,11]. The results showed dense morphology and little voids on surface in case of Cu/SnS/ZnS/Mo/glass [12,13].

There are some models to exhibit the advantageous properties of grain boundaries (GBs) of polycrystalline CIGS. Jiang et al. proposed that GBs acting as a factor to improve cell performance contrary to single-crystal solar cells by scanning probe characterization. GBs of CIGS drive majority carriers to repel out of the regime, which results in suppression of recombination [14,15]. Yan et al. suggested that GBs in CIGS electrically benign and not harmful to photovoltaic due to not creating deep levels [16]. On the other hand, valence band maximum at GBs acts as hole barriers, it reduces recombination at GBs [17]. Recently, Abou-Ras et al. identified Se-Se-terminated Σ3{112} twin boundaries, indicating that Cu is depleted and In is enriched in the two atomic planes next to the twin boundary by high-resolution scanning transmission electron microscopy and electron energy-loss spectroscopy [18]. Takahashi group in Japan also reported that downward band bending of the conduction band and broadening of the band gap near GBs are observed by photo-assisted Kelvin probe force microscopy. It accounts for photo-carriers well separate and suppress the recombination at GBs [19]. Therefore, we have to investigate carefully carrier transport at GB in CZTSSe thin films, which is not yet clearly identified for the role of GBs. We already reported positive potential bending of GBs on CZTSe thin films, grown by electron co-evaporation, which showed 2% to 3% of conversion efficiency [20]. In this study, we investigate sputtered CZTSSe thin-film solar cells, which exhibit better device performance than the previous samples. We report local carrier transport and surface potential of CZTSSe thin films using conductive atomic force microscopy (C-AFM) and Kelvin probe force microscopy (KPFM), respectively. For the complete understanding of the behaviors at GBs in CIGS films, recombination at GBs is diminished also due to downward band bending reduced density of deep-level in-gap states (i.e., recombination centers) and expect relatively efficient minority-carrier collection at GBs, as shown by scanning tunneling microscopy (STM) measurements [21,22]. Future analysis using STM can be addressed for GBs of CZTSSe thin films.

## Method

CZTSSe thin films were grown on Mo-coated soda-lime glass substrates. The metal precursor layers were deposited by radio frequency sputtering using Cu, ZnS and SnS targets. The staking order of the precursors in this study was Cu/SnS/ZnS/Mo/glass. Thickness of each stacked layer was changed from 0.4 to 0.7 μm. After the deposition, the precursors were annealed with Se metals in a furnace at 590°C for 20 min. Thickness of the annealed CZTSSe film was 1.8 μm for this study. From X-ray diffraction, the film shows single phase of CZTSSe without any significant second phases. We obtained the final composition is Cu/(Zn + Sn) ~ 0.94 and Zn/Sn ~ 1.65 of CZTSSe thin films by energy dispersive spectrometry (EDS). S/Se ratio is estimated to be approximately 0.1. The grain size indicates 1 to 2 μm of the CZTSSe thin film investigated by field emission scanning electron microscopy (FE-SEM) (JSM-700 F).

**Figure 1:**
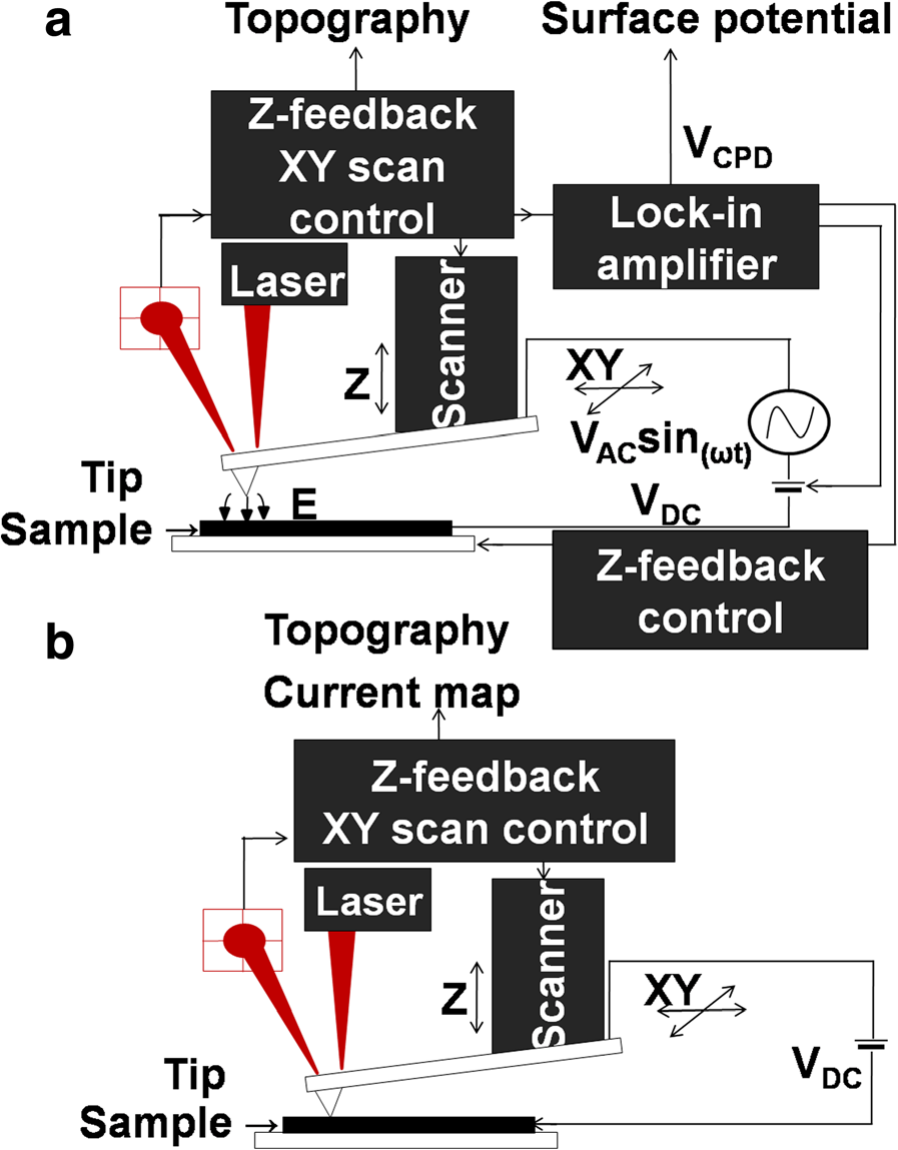
Schematic illustration of (a) Kelvin probe force microscopy and (b) conductive atomic force microscopy.

KPFM and C-AFM measurements were carried out using a commercial AFM (n-Tracer, Nanofocus Inc., Seoul, South Korea). KPFM has been used widely for metals and semiconductors to characterize electrical properties of the surfaces at nanoscale. From KPFM measurement, we obtain contact potential difference (CPD) between a metallic AFM tip and a sample which is denoted as *V*_CPD_. *V*_CPD_ can be defined as Equation (1) and identical as the work function difference between the tip and the sample if there are no defect states on the surface of the sample. If the tip approaches to the sample surface, electrostatic force is getting stronger between the tip and the sample surface. When the tip is close enough to the sample surface, Fermi levels of the tip and the sample will be aligned and become equilibrium state but the vacuum levels are not the same [23]. The external bias DC voltage (*V*_DC_) nullifies *V*_CPD_ as shown in Figure 1a. A Pt/Ir-coated tip was used for C-AFM and KPFM (Nano sensor). The surface potential and topography were determined under a non-contact mode by applying AC voltage with amplitude of 1 V (peak to peak) and frequency of 70 kHz to get clear images and sufficient sensitivity. The AC voltage will lead to an oscillating force to the tip. The feedback loop adjusted the DC potential to nullify the *V*_CPD_ component by applied DC bias to the tip, so we can obtain the two-dimensional surface potential image. The topography images were obtained by using the noncontact mode at a resonant frequency of the probe of about 73.84 kHz. The scanning rate was with 0.5 Hz to minimize topological signal and samples were not damaged performing these measurements. A lock-in amplifier was operated with a sensitivity of the 100 mV/nA.

(1) $$V_{\text{CPD}}=\frac{\varphi_{\text{tip}}-\varphi_{\text{sample}}}{-e}$$

Current maps were obtained at contact mode with applying external constant voltage 0.2 V on the samples in a 5 × 5 μm^2^ scanning areas shown in Figure 1b. The Mo layer is used for back contact which was connected to a metal-coated conducting probe that is ground. Silver paste was used for the electrical contact for this measurement. A contact force of 1 nN was applied onto a probe for the scanning area and the scanning time was set at 500 ms for each line to acquire a local current map measurement. Local current maps can be measured simultaneously together between sample and tips. The AFM laser has the wavelength of 633 nm (*E* = 1.95 eV) is above the band gap of CZTSSe films (*E* = 1.0 to 1.1 eV). Thus, the photon energy is greater than the band gap of the CZTSSe layer, the power of laser is low which does not affect photo-current contribution significantly. Considering local current and surface potential results, we can identify local electrical properties such as GBs of the CZTSSe thin film by comparing the images of the topography with that of the surface potential and current maps.

## Results and discussion

A typical device characteristic of the CZTSSe samples that are studied in this paper is summarized in Table 1. The solar cell is fabricated with *n*-doped CdS layer on the CZTSSe thin films. The CdS layer was formed by chemical bath deposition with 30 nm of thickness. Open circuit voltage (*V*_oc_) of the cell is small due to its low band gap and probably interface band-off between CdS and CZTSSe and the fill factor (FF) is relatively small because its carrier path and surface serial resistance are not defined well [24]. To obtain the high-efficiency solar cells, we need to improve *V*_oc_ and FF.

**Table 1:** Device performances and composition of CZTSSe thin-film solar cell

**Sample**	***V***_**oc **_**(mV)**	***J***_**sc **_**(mA/cm**^**2**^**)**	**F.F. (%)**	**Eff. (%)**	**Cu/Zn + Sn**	**Zn/Sn**
CZTSSe	349.00	30.61	46.13	4.93	0.94	1.65

**Figure 2:**
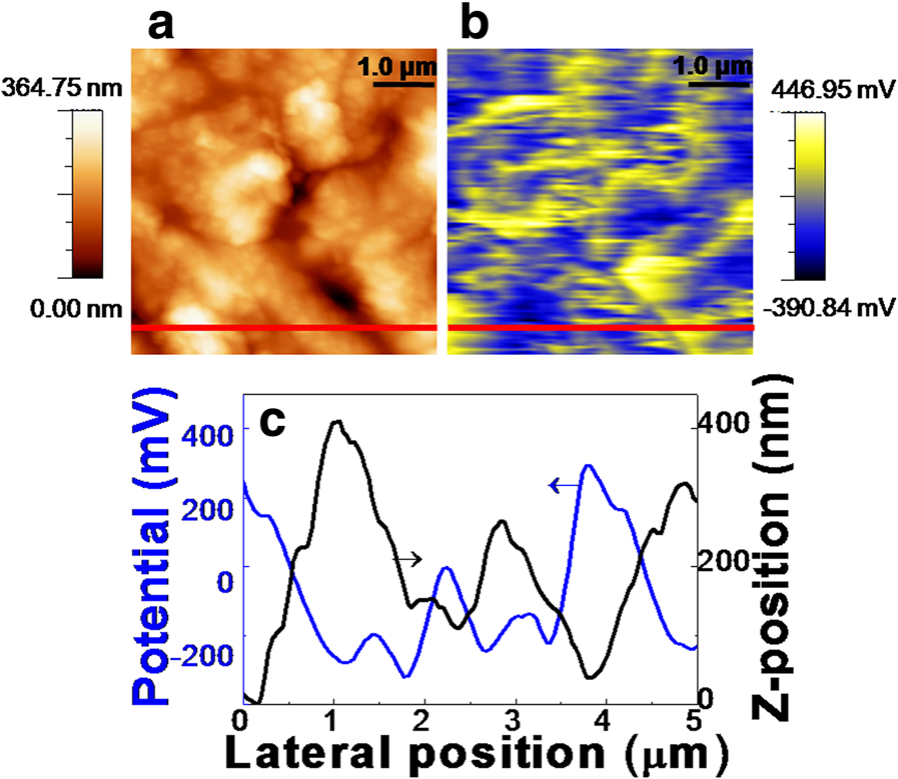
Topography (a), corresponding potential images (b), and one-dimensional line profile (c) of the CZTSSe thin film.

Figure 2 shows topography, surface potential, and the line profiles of the CZTSSe thin film. Grains of the CZTSSe films are shown in Figure 2a. The grains seem to possess small particulates. In Figure 2b, yellow region represents positive potential value and blue region indicates negative potential value. The one-dimensional line profiles in Figure 2c project the blue line of Figure 2a,b. In Figure 2c, the CZTSSe thin film reveals high positive surface potential near GBs. CIGS thin films form positively charged GBs which is related to negative band bending. The negative energy bending near GBs improves carrier separation and suppresses recombination of electron–hole pairs at GBs [14,15] because holes tend to be kept away from the GB region. However, the minority-carrier electrons are moving into the GBs, which might be a trade-off for carrier migration to the electrodes. It is desirable to study carrier transport in the intragrains (IGs) as well as the GBs. Surface potential distribution in the CZTSSe thin film shows similar behaviors to the CIGS thin films. The potential near GBs in the CZTSSe thin film indicates about 300 mV and negative potential about −100 to −200 mV at IGs, which is linked to negative band bending on GBs of the CZTSSe thin film. This is consistent with the fact that some of the minority carriers (electron) transferred to and collected at GBs in the CZTSe thin film [25]. Thus, electron–hole carriers separate effectively on GBs of CZTSSe thin film not acting as recombination center, which is a similar phenomenon occurring in CIGS. In order to clarify the relationship between topography and surface potential, we introduce a topographic parameter *Φ* = *d*^2^*H*/*dX*^2^. *H* is the height and *X* is the lateral direction. So the second derivative of *H* with respect to *X* means the concave or the convex shapes of the surface topography. Since *Φ* is an indicative of the surface alterations of the films, we can expect the positive value as GBs and the negative as IGs. From this parameter, we are able to ascertain roughly the region of GBs on the surface. Some groups claim that additional information like electron beam backscattered diffraction (EBSD) is required to confirm the granular nature of the local regions [26]. However, our approach is also widely acceptable for inspection of the surface topography and potential. The experimental results on the relationship between potential and surface are verified, but the implications of the relationship will be further investigated in particular for the regard of conversion efficiency.

### Conductive atomic force microscopy

**Figure 3:**
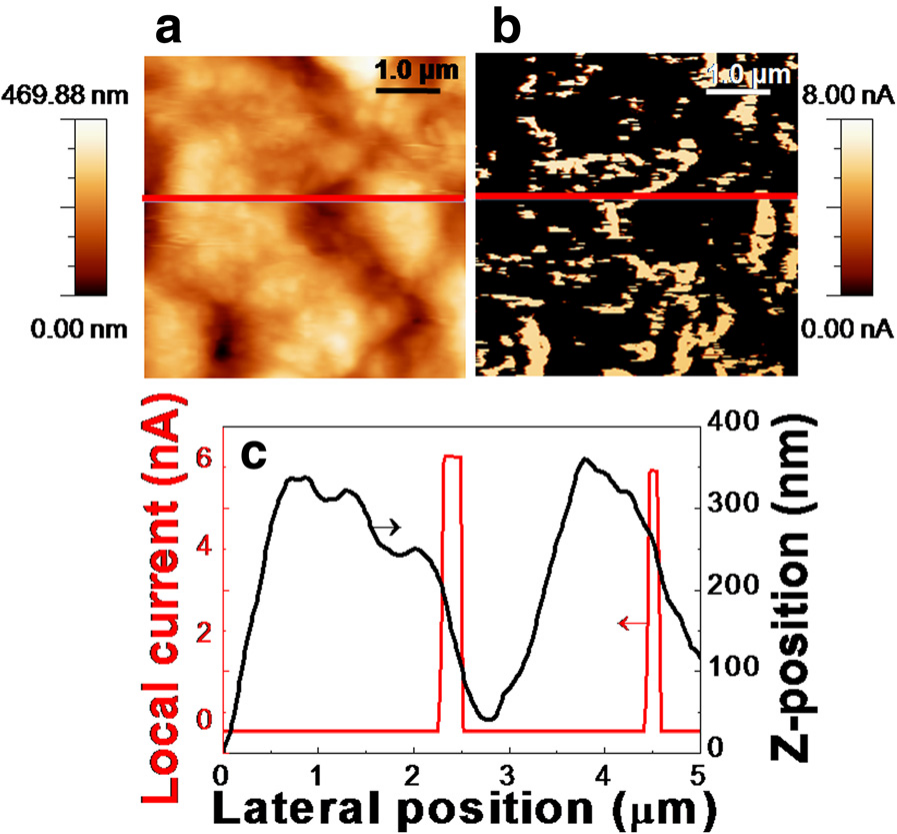
Topography (a), corresponding current-map images (b), and one-dimensional line profile (c) of the CZTSSe thin film.

Figure 3 shows topography, current map, and the line profiles of the CZTSSe thin film. Local current flows up to larger than 6 nA on GBs in the CZTSSe thin film. In case of CIGS, magnitude of current showed about 2 nA under the sample external voltage of 0.2 V [27]. The CZTSSe thin film exhibits local current flowing mostly near the GBs as displayed in Figure 3c. Local current routes are formed near the GBs of the CZTSSe thin film. The one-dimensional line profile shows the current flows at the edge of the grains. Similar current distribution was observed in the GBs of the CIGS thin films [28,29]. Azulay et al. proposed that higher dark current flow through the GBs because of higher hole mobility on the GBs and then inversion of the dominant carrier type at the GBs [29]. Therefore, electrons can become dominant carriers in GBs and drift along GBs of the CIGS thin films [27,29]. From C-AFM measurement, we can suggest that collected minority carriers form local current route through the near GBs in the case of the CZTSSe thin film, indicating that it is possible that carrier type inversion can also happen in the CZTSSe thin films.

**Figure 4:**
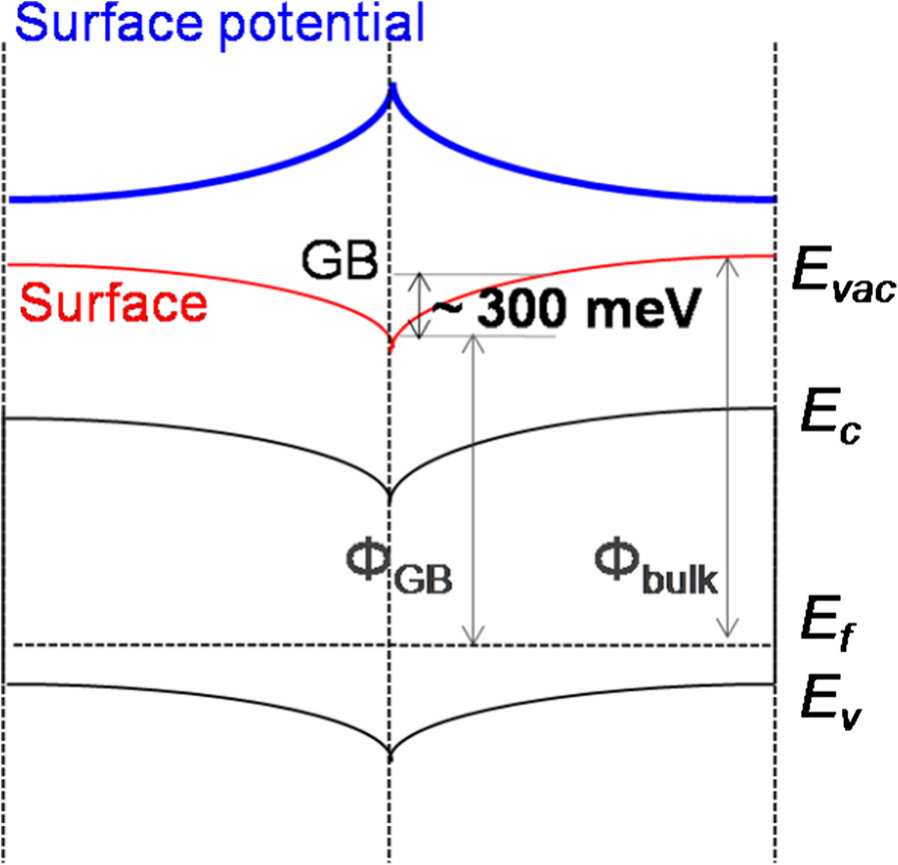
**A proposed band bending near the GBs of the CZTSSe thin films.** The band diagram also accounts for the minority carrier transport near the GBs.

From the measurement results of KPFM and C-AFM, we found positive potential on the most of GBs and demonstrated downward band bending in the CZTSSe thin film. On the other hand, the negative potential on the GBs is linked to the upward band bending. A model of surface potential and carrier transport is described in Figure 4. The positively charged GBs play a role to be a conduction path and collect minority carriers. However, the defects in the GBs are not well known yet. So, the carriers can be trapped in the defects near the GBs [30], which may be drawbacks for high efficiency of the CZTSSe solar cells. The model of band diagram depends on charged GBs can be affected by film properties such as composition and conversion efficiency [20]. It is indispensible to understand the defect chemistry and transport near GBs of the CZTSSe. If all the understandings are well established and proper processing methods are developed, polycrystalline kesterite thin films are beneficial to device performance for solar cells.

## Conclusions

We measured surface potential and current transport of the CZTSSe thin film with Kelvin probe force microscopy and conductive atomic force microscopy, respectively. For these studies can demonstrate the essential of combining local electrical characterization techniques to investigate the local current and potential properties of kesterite materials. The surface potential near GBs shows negative band bending behaviors with about 300 meV of energy shift. In the current map, the dominant current flow path is observed through GBs, which is governed by minority carriers. Most of the electrical properties of the CZTSSe are very similar to the CIGS, but we will study more the details to explain the physical and chemical properties in the interface of the CZTSSe thin films for high conversion efficiency.

## Abbreviations

C-AFM: Conductive atomic force microscopy; CIGS: Cu(In,Ga)Se_2_; CZTSSe: Cu_2_(Zn,Sn)(S,Se)_4_; EBSD: Electron beam backscattered diffraction; Eff.: Photo-conversion efficiency; F.F.: Fill factor; GBs: Grain boundaries; IGs: Intragrains; Jsc: Short circuit current; KPFM: Kelvin probe force microscopy; Voc: Open circuit voltage.

## Competing interests

The authors declare that they have no competing interests.

## Authors’ contributions

GYK, JRK, and WJ measured the electrical properties of the CZTSSe samples with scanning probe microscopy. DHS, DHK, and JKK made the CZTSSe samples by sputtering and subsequent selenization. All authors read and approved the final manuscript.

## References

[B1] Chen S Gong XG Walsh A Wei S-H Electronic structure and stability of quaternary chalcogenide semiconductors derived from cation cross-substitution of II-VI and I-III-VI_2_ compounds Phys Rev B 2009 9 165211

[B2] Todorov TK Tang J Bag S Gunawan O Gokmen T Zhu Y Mitzi DB Beyond 11% efficiency: characteristics of state-of-the-art Cu_2_ZnSn(S, Se)_4 _solar cells. Adv Energy Mater 2013 9 34 38 10.1002/aenm.201200348

[B3] W-C H Repins I Beall C DeHart C To B Yang W Yang Y Noufi R Growth mechanisms of co-evaporated kesterite: a comparison of Cu-rich and Zn-rich composition paths Prog Photovolt: Res Appl 2014 9 35 43 10.1002/pip.2296

[B4] Repins I Beall C Vora N DeHart C Kuciauskas D Dippo P To B Mann J W-C H Goodrich A Noufi R Co-evaporated Cu_2_ZnSnSe_4_ films and devices Sol Energy Mater Sol Cells 2012 9 154 159

[B5] Hiroi H Sakai N Kato T Sugimoto H High voltage Cu_2_ZnSnS_4_ submodules by hybrid buffer layer Proceedings of the IEEE Photovoltaic Specialists Conference 39th: 16–21 June 2013 Tampa, FL

[B6] Katagiri H Jimbo K Maw WS Oishi K Yamazaki M Araki H Takeuchi A Development of CZTS-based thin film solar cells Thin Solid Films 2009 9 2455 2460 10.1016/j.tsf.2008.11.002

[B7] Shin SW Pawar SM Park CY Yun JH Moon J-H Kim JH Lee JY Studies on Cu_2_ZnSnS_4_ (CZTS) absorber layer using different stacking orders in precursor thin films Sol Energy Mater Sol Cells 2011 9 3202 3206 10.1016/j.solmat.2011.07.005

[B8] Zoppi G Forbes I Miles RW Dale PJ Scragg JJ Peter LM Cu_2_ZnSnSe_4_ thin film solar cells produced by selenization of magnetron sputtered precursors Prog Photovolt: Res Appl 2009 9 315 319 10.1002/pip.886

[B9] Scragg JJ Ericson T Fontané X Izqierdo-Roca V Pérez-Rodríguez A Kubart T Edoff M Platze-Björkman C Rapid annealing of reactively sputtered precursors for Cu_2_ZnSnS_4_ solar cells Prog Photovolt: Res Appl 2014 9 10 17 10.1002/pip.2265

[B10] Momose N Htay MT Yudasaka T Igarashi S Seki T Iwano S Hashimoto Y Ito K Cu_2_ZnSnS_4_ thin film solar cells utilizing sulfurization of metallic precursor prepared by simultaneous sputtering of metal targets Jpn J Appl Phys 2011 9 01BG09 10.7567/JJAP.50.01BG09

[B11] Araki H Kubo Y Mikaduki A Jimbo K Maw WS Katagiri H Yamazaki M Oishi K Takeuchi A Preparation of Cu_2_ZnSnS_4_ thin films by sulfurizing electroplated precursors Sol Energy Mater Sol Cells 2009 9 996 999 10.1016/j.solmat.2008.11.045

[B12] Jimbo K Kimura R Kamimura T Yamada S Maw WS Araki H Oishi K Katagiri H Cu_2_ZnSnS_4_-type thin film solar cells using abundant materials Thin Solid Films 2007 9 5997 5999 10.1016/j.tsf.2006.12.103

[B13] Jackson P Hariskos D Lotter E Paetel S Wuerz R Menner R Wischmann W Powalla M New world record efficiency for Cu(In, Ga)Se_2_ thin-film solar cells beyond 20% Prog Photovolt: Res Appl 2011 9 894 897 10.1002/pip.1078

[B14] Jiang C-S Noufi R AbuShama JA Ramanathan K Moutinho HR Pankow J Al-Jassim MM Local built-in potential on grain boundary of Cu(In, Ga)Se_2_ thin film Appl Phys Lett 2004 9 3477 3479 10.1063/1.1737796

[B15] Jiang C-S Noufi R Ramanathan K AbuShama JA Moutinho HR Al-Jassim MM Does the local built-in potential on grain boundaries of Cu(In, Ga)Se_2_ thin films benefit photovoltaic performance of the device? Appl Phys Lett 2004 9 2625 2627 10.1063/1.1793346

[B16] Yan Y Jiang C-S S–H W Moutinho HR Al-Jassim MM Electrically benign behavior of grain boundaries in polycrystalline CuInSe_2_ Films Phys Rev Lett 2007 9 235504 18233382 10.1103/PhysRevLett.99.235504

[B17] Persson C Zunger A Compositionally induced valence-band offset at the grain boundary of polycrystalline chalcopyrites creates a hole barrier Appl Phys Lett 2005 9 211904 10.1063/1.2132537

[B18] Abou-Ras D Schaffer B Schaffer M Schmidt SS Caballero R Unold T Direct insight into grain boundary reconstruction in polycrystalline Cu(In, Ga)Se_2_ with atomic resolution Phys Rev Lett 2012 9 075502 22401224 10.1103/PhysRevLett.108.075502

[B19] Takihara M Minemoto T Wakisaka Y Takahashi T An investigation of band profile around the grain boundary of Cu(In,Ga)Se_2_ solar cell material by scanning probe microscopy Prog Photovolt: Res Appl 2013 9 595 599

[B20] Jeong AR Jo W Jung S Gwak J Yun JH Enhanced exciton separation through negative energy band bending at grain boundaries of Cu_2_ZnSnSe_4_ thin-films Appl Phys Lett 2011 9 082103 10.1063/1.3626848

[B21] Mönig H Smith Y Caballero R Kaufmann CA Lauermann I Lux-Steiner MC Sadewasser S Direct evidence for a reduced density of deep level defects at grain boundaries of Cu(In, Ga)Se_2_ thin films Phys Rev Lett 2010 9 116802 20867594 10.1103/PhysRevLett.105.116802

[B22] Azulay D Balberg I Millio O Microscopic evidence for the modification of the electronic structure at grain boundaries of Cu(In_1-x_, Gax)Se_2 _films. Phys Rev Lett 2012 9 076603 22401233 10.1103/PhysRevLett.108.076603

[B23] Melitz W Shen J Kummel AC Lee S Kelvin probe force microscopy and its application Surf Sci Rep 2011 9 1 27 10.1016/j.surfrep.2010.10.001

[B24] Guo Q Ford GM Yang W-C Walker BC Stach EA Hillhouse HW Agrawal R Fabrication of 7.2% efficient CZTSSe solar cells using CZTS nanocrystals J AM CHEM SOC 2010 9 17384 17386 10.1021/ja108427b 21090644

[B25] Li JB Chawla V Clemens BM Investigating the role of grain boundaries in CZTS and CZTSSe thin film solar cells with scanning probe microscopy Adv Mater 2012 9 720 723 10.1002/adma.201103470 22228331

[B26] Sadewasser S Abou-Ras D Azulay D Baier R Balberg I Cahen D Cohen S Gartsman K Ganesan K Kavalakkatt J Li W Millo O Rissom T Rosenwaks Y Schock H-W Schwarzman A Unold T Nanometer-scale electronic and microstructural properties of grain boundaries in Cu(In, Ga)Se_2_ Thin Solid Films 2011 9 7341 7346 10.1016/j.tsf.2010.12.227

[B27] Shin RH Jo W Kim D-W Yun JH Ahn S Local current–voltage behaviors of preferentially and randomly textured Cu(In, Ga)Se_2_ thin films investigated by conductive atomic force microscopy Appl Phys A 2011 9 1189 1194 10.1007/s00339-011-6408-y

[B28] Shin RH Jeong AR Jo W Investigation of local electronic transport and surface potential distribution of Cu(In, Ga)Se_2_ thin-films Curr Appl Phys 2012 9 1313 1318 10.1016/j.cap.2012.03.016

[B29] Azulay D Millo O Balberg I Schock HW Visoly-Fisher I Cahen D Current routes in polycrystalline CuInSe_2_ and Cu(In, Ga)Se_2_ films Sol Energy Mater Sol Cells 2007 9 85 90 10.1016/j.solmat.2006.08.006

[B30] Li J Mitzi DB Shenoy VB Structure and electronic properties of grain boundaries in earth-abundant photovoltaic absorber Cu_2_ZnSnSe_4_ ACS Nano 2011 9 8613 8619 10.1021/nn203230g 22007834

